# Mesenchymal stem cells derived exosomes and microparticles protect cartilage and bone from degradation in osteoarthritis

**DOI:** 10.1038/s41598-017-15376-8

**Published:** 2017-11-24

**Authors:** Stella Cosenza, Maxime Ruiz, Karine Toupet, Christian Jorgensen, Danièle Noël

**Affiliations:** 1 IRMB, INSERM, Montpellier University, Montpellier, France; 2 Clinical immunology and osteoarticular diseases Therapeutic Unit, Hôpital Lapeyronie, Montpellier, France

## Abstract

Mesenchymal stem or stromal cells (MSCs) exert chondroprotective effects in preclinical models of osteoarthritis (OA). Most of their therapeutic effects are mediated via soluble mediators, which can be conveyed within extracellular vesicles (EVs). The objective of the study was to compare the respective role of exosomes (Exos) or microvesicles/microparticles (MPs) in OA. MPs and Exos were isolated from bone marrow murine BM-MSCs through differential centrifugation. Effect of MPs or Exos was evaluated on OA-like murine chondrocytes and chondroprotection was quantified by RT-qPCR. In OA-like chondrocytes, BM-MSC-derived MPs and Exos could reinduce the expression of chondrocyte markers (type II collagen, aggrecan) while inhibiting catabolic (MMP-13, ADAMTS5) and inflammatory (iNOS) markers. Exos and MPs were also shown to protect chondrocytes from apoptosis and to inhibit macrophage activation. *In vivo*, Exos or MPs were injected in the collagenase-induced OA (CIOA) model and histomorphometric analyses of joints were performed by µCT and confocal laser microscopy. BM-MSCs, MPs and Exos equally protected mice from joint damage. In conclusion, MPs and Exos exerted similar chondroprotective and anti-inflammatory function *in vitro* and protected mice from developing OA *in vivo*, suggesting that either Exos or MPs reproduced the main therapeutic effect of BM-MSCs.

## Introduction

Osteoarthritis (OA) is the most prevalent rheumatic disease, characterized by cartilage degradation, sub-chondral bone sclerosis, osteophyte formation, synovial inflammation and calcification of ligaments. The main risks factors are age, obesity, genetics and joint injuries following traumas. Its prevalence is constantly increasing, making this disease among the main causes of years lived with disability^1^. It induces enormous public health resources devoted to the cure, prevention and amelioration of sequelae of the disease. Current treatments are only symptomatic relying on the use of antalgics and non-steroid anti-inflammatory drugs (NSAID). Because these treatments are palliative and not curative, other therapeutic strategies have to be developed. In recent years, mesenchymal stem/stromal cell (MSC)-based therapies have emerged as a novel opportunity to positively impact on the outcome of OA. We and others have shown in pre-clinical models that intra-articular injection of MSCs, either from bone marrow or adipose tissue, could protect cartilage from degeneration and at least delay OA progression^2–5^. MSC-based therapy for OA is also being evaluated in the clinics and encouraging results on pain and inflammation reduction have been reported^6,7^. Most of the therapeutic effects are attributed to the secretion of mediators with anti-inflammatory and chondroprotective functions^8,9^. However, some recent evidence suggests that those mediators are conveyed within extracellular vesicles (EVs) released by MSCs.

EVs are a heterogeneous population of particles released by virtually all cell types and involved in cell-to-cell communication pathways. There are three main classes: exosomes or small-size vesicles, microparticles or microvesicles and apoptotic bodies. They are characterized by their size, biogenesis and expression of membrane markers. Exosomes (Exos) are generated via the endosomal compartment in multivesicular bodies and express endosomal markers (CD9, CD61, CD83, ALIX, TSG101) while microparticles (MPs) are released by cell membrane budding and express markers from the parental cell (for review, see^10^). The therapeutic effect of MSC-derived EVs in OA has been recently described in three studies with contradictory results. In the first one, MSC-derived Exos were shown to decrease the anabolic function of chondrocytes, except when MSCs were engineered to express miR-140-5p^11^. In the second one, Exos isolated from iPS-derived MSCs or synovial MSCs successfully decreased OA symptoms *in vivo* but Exos from iPS-derived MSCs were more efficient^12^. In the most recent article, a beneficial effect of embryonic stem cell-derived Exos was reported in the destabilization of the medial meniscus (DMM) model^13^. However, none of these studies reported the effect of other types of EVs. One objective of the present study was to characterize *in vitro* the functional role of either Exos or MPs isolated from bone marrow (BM-MSCs) on the function of cells from the articular environment, chondrocytes and monocytes/macrophages. The second objective was to characterize in depth the *in vivo* therapeutic effect of the two types of EVs in a preclinical model of OA using quantitative histomorphometric parameters of bone and cartilage tissues.

## Materials and Methods

### Mesenchymal stem cell culture and EV production

Murine BM-MSCs were isolated from bone marrow of C57BL/6 mice and previously characterized by phenotyping and trilineage differentiation potential as described in^14^. They were expanded in proliferative medium consisting in DMEM, 100 µg/mL penicillin/streptomycin, 2 mmol/mL glutamine and supplemented with 10% foetal calf serum (FCS). BM-MSCs were used between passages 10 and 20.

For EV production, BM-MSCs were seeded in proliferative medium at 2 × 10^4^ cells/cm² and maintained in proliferative medium for 24 h. For evaluating the chondroprotective function of EVs uniquely, TGF-β3 (10 ng/mL) was added in the proliferative medium for 24 h. Proliferative medium was then replaced by production medium consisting in DMEM, 100 µg/mL penicillin/streptomycin, 2 mmol/mL glutamine and 3% EVs-free FCS. EVs-free FCS containing medium was obtained by ultracentrifugation of DMEM plus 20% FCS at 100,000 g overnight and kept at 4 °C before dilution with DMEM for use. After 48 h, BM-MSC-conditioned medium (CM) was centrifuged at 300 g for 10 min to eliminate cells and 2,500 g for 25 min to remove debris and apoptotic bodies. For MP isolation, CM was centrifuged at 18,000 g for 1 h in polyallomer tubes; the pellet was then suspended in PBS and submitted to a second round of centrifugation. For Exos, supernatant from MP fraction was filtered on 0.22 µm porous membrane and centrifuged at 100,000 g for 2 h. Pellet was suspended in PBS and centrifuged again at 100,000 g for 2 h. Both MP and Exo pellets were suspended in 100 µL of PBS and freshly used for *in vitro* and *in vivo* functional experiments.

### EV characterization

Production of EVs was normalized to the content in total protein as quantified by Bradford Colorimetric Assay (BCA) assay. Size distribution of EVs was determined by Nanoparticle Tracking Analysis in a NanoSight LM10-12 instrument as advised by manufacturer (Malvern) and by Dynamic Light Scattering (DLS).

### ***In vitro*** model of OA like chondrocytes

Murine chondrocytes were isolated from 3 days old C57BL/6 mice as described in^15^ and, induced to express an OA-like phenotype by addition of IL-1β as described elsewhere (Ruiz *et al*., submitted). Briefly, IL-1β (1 ng/mL) was added to chondrocytes cultured in DMEM containing 10% FCS, 100 µg/mL penicillin/streptomycin, 2 mmol/mL glutamine. In parallel, BM-MSC-CM was prepared from BM-MSCs cultured in proliferative medium supplemented or not with 10 ng/mL TGF-β3. After 24 h, chondrocyte medium was replaced by medium containing different amounts of MPs or Exos (12.5 ng; 125 ng or 1.25 µg), BM-MSC-CM (1 mL) or BM-MSCs (10^5^ cells) on top of a transwell membrane. Following another 24 h of incubation, chondrocytes were then recovered and processed for RT-qPCR analysis.

### Apoptosis induction

Murine articular chondrocytes were isolated and plated in 12 wells culture plates as described above. After 5 days, confluent BM-MSCs (10^5^ cells) adherent on a transwell membrane (0.4 µm) were added in chondrocytes-containing wells for 24 h. Afterwards, all media in wells containing chondrocytes alone or chondrocytes/BM-MSCs cocultures were replaced by fresh medium containing staurosporine (150 ng in 1 mL/well). At the same time, two doses of MPs or Exos (125 ng or 250 ng) were added to the wells containing chondrocytes alone. After 6 hours, cells were trypsinized and labelled for flow cytometry analysis.

### Macrophage isolation and differentiation

Macrophages were isolated from spleens using the positive selection CD11b kit as recommended (Miltenyi, Paris, France). CD11b^+^ cells (2 × 10^5^ cells/cm^2^) were activated by lipopolysaccharides (LPS) as described in^16^. When indicated, BM-MSCs (ratio 1BM-MSC/5cells) or 50 ng of MPs or Exos were added for 3 days. Cells were recovered for flow cytometry analysis and supernatants for cytokine quantification by ELISA.

### Flow cytometry analysis

For EVs, suspensions of MPs or Exos (1 µg equivalent proteins) were coated onto 4 µm aldehyde/sulfate latex beads by incubation at 4 °C overnight and free reactive sites on beads were filled by adding 100 mM glycine. Beads coated with EVs were then washed 3 times in PBS and 1 µL of specific antibodies for CD9 (clone MZ3, Miltenyi Biotec), CD29 (clone Ha2/5, BD Biosciences), CD44 (clone IM7, BD Biosciences), CD81 (clone EAT2, Miltenyi Biotec), SCA-1 (clone D7, BD Biosciences) were added for 30 min.

For macrophage analysis, cells were suspended in PBS supplemented with 0.2% bovine serum albumin (BSA) and incubated with antibodies specific for F4/80 (clone BM8, eBiosciences), MHCII (clone MS/114.15.2, Miltenyi Biotec), CD40 (clone 3/23, BD Biosciences), CD80 (clone 16-10A1, BD Biosciences), CD86 (clone GL1, BD Biosciences), or respective isotype controls at 4 °C for 20 min. For apoptosis detection, cells were incubated with the Annexin V-PE apoptosis detection kit following manufacturer’s instructions (eBioscience). Data acquisition was performed by flow cytometry using a FACSCanto cytometer and analysis of data was done with Diva software.

### Collagenase-induced arthritis model

Collagenase-induced OA (CIOA) model was performed as previously described^17^ and in accordance with guidelines and regulations of the Ethical Committee for animal experimentation of the Languedoc-Roussillon (Approval 5349-2016050918198875). All experiments were performed after final approval given by the French Ministry for Education, Higher Education and Research. Briefly, 1U type VII collagenase in 5 µL saline was administered intra-articularly (IA) in the knee joint of 10 weeks old C57BL/6 mice at day 0 and 2. Groups of 15 mice received IA injections of either BM-MSCs (2.5 × 10^5^ cells/5 µL saline), MPs (500 ng/5 µL) or Exos (250 ng/5 µL) at day 7. Mice were euthanatized at day 42 and paws recovered for fixation in 4% formaldehyde and further analysis.

### Bone parameter analyses

Hind paws were dissected to carefully remove smooth tissues and scanned in a microCT scanner SkyScan 1176 (Bruker, Belgium, 0.5 mm aluminium filter, 45 kV, 500 µA, resolution of 18 µm, 0.5° rotation angle). Scans were reconstructed using NRecon software (Bruker, Belgium). Misalignment compensation, ring artifacts and beam-hardening were adjusted to obtain a correct reconstruction of each paw. Bone degradation was quantified in subchondral bone and epiphysis region of medial plateau for each tibia (CTAn software, Bruker, Belgium). Osteophyte formation on joint edges and meniscal/ligament calcification were quantified on the entire knee joint. Reconstructed 3D images of joints were obtained using Avizo software (Avizo Lite 9.3.0, FEI, France).

### Confocal laser scanning microscopy

Articular cartilage of tibia medial plateau was scanned through their depth in XYZ-mode, with a confocal laser scanning microscope (CLSM; TCS SP5-II, Leica Microsystems, Nanterre, France) with a voxel size of 6 µm, a 5× dry objective and a UVlaser light source (l¼ 405 nm). Stacks of images were then done and analyzed to quantitatively evaluate several parameters of articular cartilage. Assessment of cartilage morphometric parameters was performed in medial plateau of each tibia using Avizo software (FEI Visualization Sciences Group, Lyon).

### Histological analysis

Hind paws were decalcified using a solution of formic acid 5% for 2 weeks and then embedded in paraffin. Frontal sections of tibias were cut (3 slices of 7 µm each 100 µm; first section at 50 µm below the cartilage surface) and stained with safranin O fast green staining. Cartilage degradation was quantified on medial plateau using the modified Pritzker OARSI score as described (Table 1) and^3^. Osteophyte size at the edges of tibia cartilage was scored using an arbitrary score from 0 to 3 as described^2^.

**Table 1:** OA score from modified Pritzker OARSI score.

Grade	Observation
**0**	normal
**1**	irregular but intact surface
**2**	superficial fibrillation
**3**	vertical fissures
**4**	partial loss of cartilage not extending to the tidemark
**5**	partial loss of cartilage beyond the tidemark but not till bone
**6**	bone loss, remodeling, deformation
**Stade**	**Observation**
**0**	No OA
**1**	OA signs < 10% of the surface
**2**	OA signs: 10–25% of the surface
**3**	OA signs: 25–50% of the surface
**4**	OA signs: 50–75% of the surface
**5**	OA signs > 75% of the surface

Final score = Grade X Stade (0 to 30).

### Statistical analyses

Statistical analysis was performed with GraphPad 6 Prism Software. Data were compared using the Mann-Whitney’s test for nonparametric values (*in vitro* experiments) or a student’s t test for animal experimentation (n = 15/group). A p value < 0.05 was considered significant.

## Results

### Isolation and characterization of MPs and Exos from BM-MSC-conditioned medium

**Figure 1:**
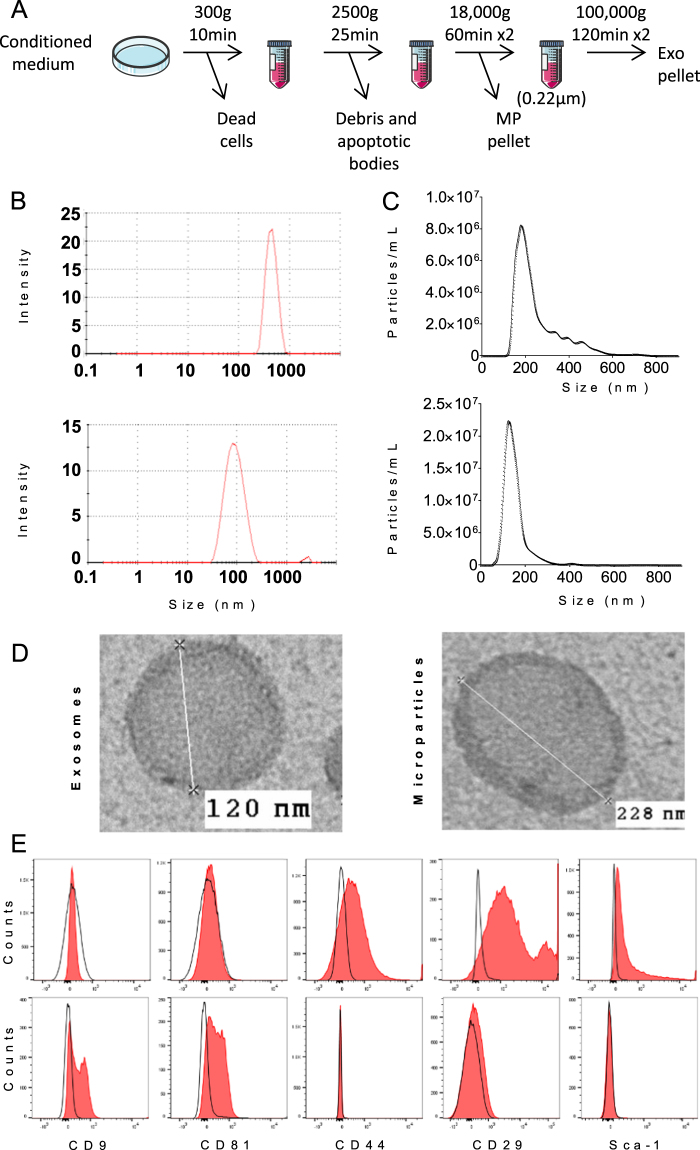
Isolation and characterization of extracellular vesicles isolated from murine BM-MSCs. (**A**) Experimental protocol for isolation of microparticles (MPs) and exosomes (Exos) using differential ultracentrifugation. (**B**) Size of MPs (up) and Exos (down) detected in 200 µL by Dynamic Light Scattering analysis (**C**) Number and size of MPs (up) and Exos (down) detected in 1 mL (corresponding to 1 µg EV equivalent proteins) by Nano Tracking Analysis. (**D**) Representative images of Exos and MPs by transmission electron microscopy. (**E**) Expression of BM-MSC membrane markers (Sca-1, CD44, CD29) and of exosomal markers (CD9, CD81) on MPs (top) and Exos (bottom) isolated from naïve BM-MSCs as analysed by flow cytometry.

MPs and Exos were isolated from 48h-conditioned medium of bone marrow-derived murine BM-MSCs. MPs-containing pellets were isolated by a centrifugation step at 18,000 g while Exos were recovered from MP-deprived supernatants filtered onto a 0.22 µm membrane and centrifuged at 100,000 g (Fig. 1A). Size of both EV preparations was measured by DLS and found to peak at 488 nm for MPs and 96 nm for Exos (Fig. 1B). To check size homogeneity of EV populations, we then performed Nano Tracking Analysis and confirmed a homogeneous population of Exos whose size was 112 ± 6.6 nm (Fig. 1C). However for MPs, a heterogeneous population of particles was observed ranging from 150 to 600 nm; size of the majority of particles being 223 ± 15.6 nm. Membrane marker profile identified expression of the BM-MSC markers CD29, CD44 and Sca-1 on MPs while endosomal markers were not detected (Fig. 1D). By contrast, the endosomal markers CD9, CD81 were expressed on Exos but membrane markers of BM-MSCs were absent. These data indicated an enrichment of MPs and Exos by centrifugation at 18,000 g and 100,000 g respectively, supporting the feasibility to investigate the respective role of MPs and Exos in the following experiments.

### Both MPs and Exos restored the anabolic/catabolic equilibrium in OA-like chondrocytes

To investigate a possible role of BM-MSC-derived MPs and Exos in OA, we first evaluated *in vitro* their capacity to restore cartilage homeostasis. We relied on a model of OA-like murine chondrocytes used in our laboratory (Ruiz, manuscript submitted). In this model, incubation of chondrocytes with IL-1β resulted in down-regulation of the anabolic marker genes COL2B, ACAN, COL1 and up-regulation of catabolic MMP-13, ADAMTS5 and inflammatory iNOS marker genes (Supl. Figure 1). Addition of BM-MSCs cultured on a transwell membrane, or conditioned medium from BM-MSCs (BM-MSC-CM), induced the expression of ACAN and COL2B genes while it reduced expression of iNOS and MMP-13. BM-MSCs cocultured in transwell did not greatly change the chondrocyte expression profile. In these conditions, addition of different amounts of MPs or Exos partly reproduced the effect of BM-MSC-CM but without a clear dose-dependent effect.

**Figure 2:**
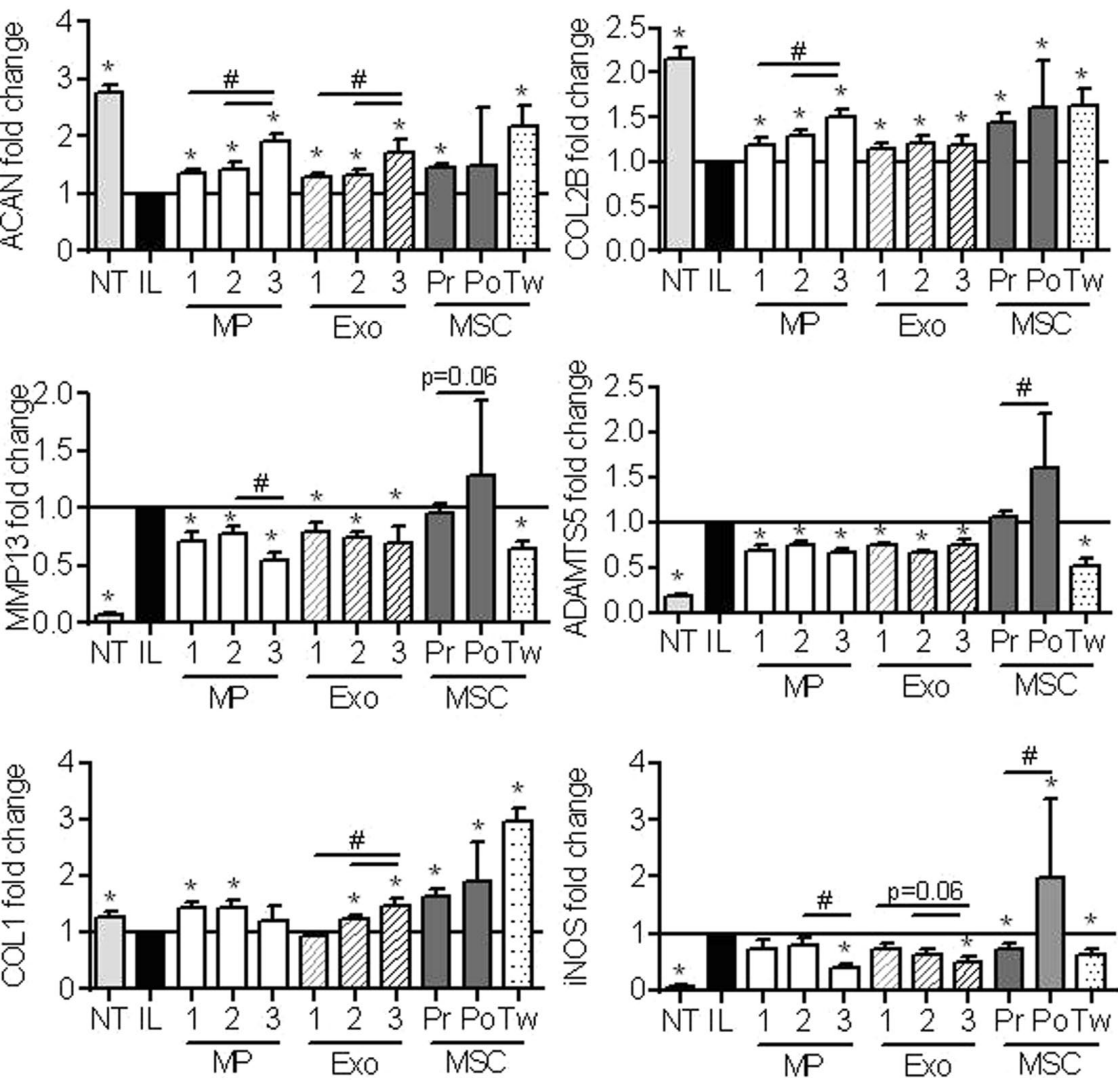
MPs and Exos isolated from TGFβ3-pretreated BM-MSCs exerted high chondroprotective effect on OA-like chondrocytes. Primary murine chondrocytes were pretreated with 1 ng/mL IL-1β (IL) or not (NT) for 24 h before addition of different amounts of MPs or Exos (1: 12.5 ng; 2: 125 ng; 3: 1.25 µg), 1 mL TGFβ3-pretreated pre-centrifugation (Pr) or post-ultracentrifugation (Po) BM-MSC-CM or TGFβ3-pretreated BM-MSCs (10^5^ cells) on top of a transwell membrane (Tw). Expression of chondrocyte markers was quantified by RT-qPCR after 24h (n = 10). *: p < 0.05 as compared to IL1-β-treated OA-like chondrocytes; #: p < 0.05 as compared to indicated groups.

Because TGF-β is a potent inducer of chondrocyte anabolism, we evaluated the effect of TGF-β3 pre-activation of BM-MSCs in this assay. Pre-centrifugation BM-MSC-CM (Pr) increased the expression of anabolic marker genes (ACAN, COL1, COL2B) and decreased iNOS expression (Fig. 2). However, the post-centrifugation BM-MSC-CM (Po) was not efficient on ACAN, MMP13, ADAMTS5, iNOS expression and significantly different from Pr BM-MSC-CM for 3 out of 6 markers. As expected, BM-MSCs in coculture (Tw) significantly decreased the expression of catabolic and inflammatory marker genes MMP-13, ADAMTS5, iNOS and increased ACAN, COL2B, COL1 expression (Fig. 2). Interestingly, addition of both MPs and Exos greatly enhanced the expression of anabolic markers in a dose-dependent manner and down-regulated that of catabolic marker genes. Supply of the highest dose of MPs and Exos exerted similar modulation on anabolic and catabolic chondrocyte marker genes as BM-MSCs in coculture. BM-MSC-derived MPs and Exos reproduced to a large extent the anabolic and chondroprotective effect of BM-MSCs.

### Both MPs and Exos exerted an anti-apoptotic effect on OA-like chondrocytes and inhibited macrophage differentiation

**Figure 3:**
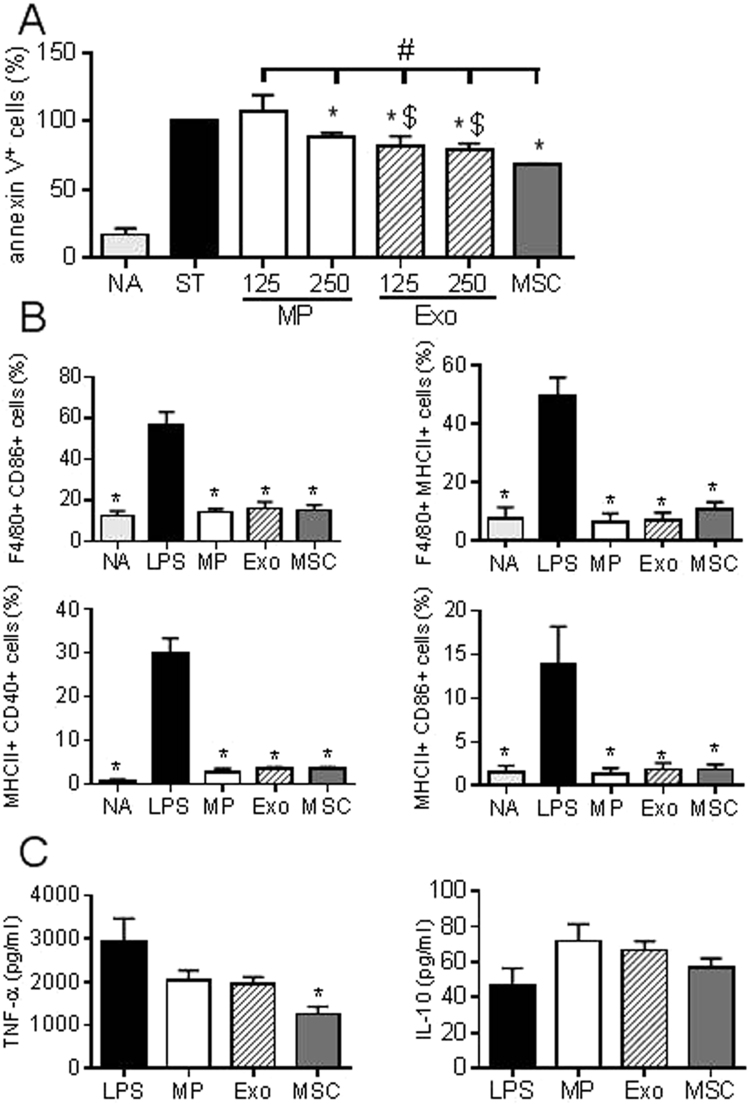
BM-MSC-derived MPs and Exos exerted anti-apoptotic effect on chondrocytes and immunosuppressive function on macrophages. (**A**) Percentage of annexin V^+^ apoptotic chondrocytes under non activated conditions (NA), staurosporine–induced apoptosis (ST) and cultured with Exos or MPs (125 ng or 250 ng) or 1 mL BM-MSC-conditioned medium (n = 5). *: p < 0.05 as compared to ST group, #: p < 0.05 as compared to BM-MSC group or $: p<0.05 s compared to MP group of same amount. (**B**) Expression of differentiation markers on non-activated CD11b^+^ macrophages (NA), on macrophages after LPS-induced activation cultured alone (LPS) or with 50 ng MPs, Exos or BM-MSCs (1 BM-MSC/5 macrophages) for 3 days (n = 4). (**C**) Quantification of cytokines produced by macrophages after 3 days as described in (B). *: p < 0.05 as compared to LPS.

A characteristic of OA cartilage is enhanced apoptosis. We therefore investigated the impact of MPs and Exos on apoptosis induction in chondrocytes. We used a model of staurosporine-induced apoptosis previously described^8^. Coculture of BM-MSCs with murine chondrocytes prevented apoptosis and reduced the percentage of apoptotic chondrocytes to 68% (Fig. 3A). Similarly, MPs and Exos reduced in a dose-dependent manner the level of apoptotic chondrocytes but Exos were more efficient than MPs. In addition, the anti-apoptotic activity of MPs and Exos, at the tested doses, was significantly lower than that of BM-MSCs.

Another feature of OA is synovial inflammation, notably characterized by activation of monocytes and macrophages. One major immunosuppressive effect of BM-MSCs is to inhibit macrophage activation and to induce a shift from M1 pro-inflammatory to M2 anti-inflammatory phenotype^18^. We therefore activated spleen-derived macrophages by LPS and investigated expression of activation markers. Addition of BM-MSCs, MPs or Exos resulted in high inhibition of macrophage activation, as shown by low percentages of F4/80^+^ macrophages expressing CD86, MHCII or CD40 markers (Fig. 3B). Reduced activation of macrophages was confirmed by down-regulation of TNF-α and up-regulation of IL-10; although TNF-α was only significantly reduced by BM-MSCs (Fig. 3C). Indeed, both MPs, Exos and BM-MSCs inhibited *in vitro* macrophage activation to a similar extent.

### MPs and Exos are both potent to protect cartilage and bone from degradation in the collagenase-induced OA murine model

**Figure 4:**
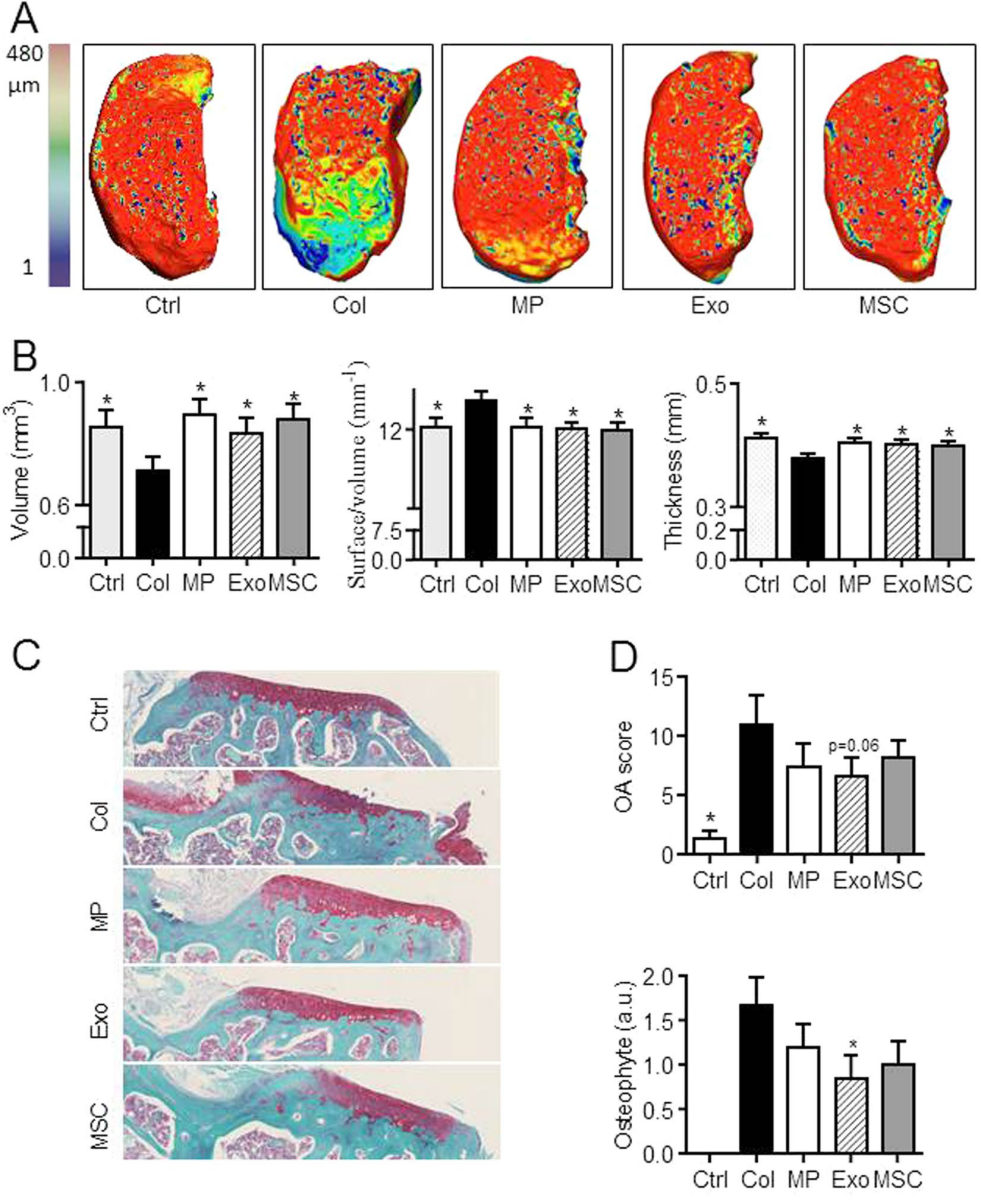
BM-MSC-derived MPs and Exos protected mice from osteoarthritic damages in the collagenase-induced OA model. (**A**) Representative 3D reconstructed images of articular cartilage after confocal laser scanning microscopy analysis. Images from control mice (Ctrl), collagenase-treated mice (Col) and Col mice that received intra-articular injection of 500 ng MPs or 250 ng Exos or 2.5 × 10^5^ BM-MSCs. (**B**) Histomorphometric analysis of 3D images of articular cartilages as described in (A) (n = 15). (**C**) Representative histological sections of tibias from mice described in (**A**) after Safranin O-Fast green staining. (**D**) OA score and osteophyte score expressed as arbitrary unit (a.u.) on histological sections of mice. *: p < 0.05 as compared to Col group.

We next aimed at evaluating whether MPs and Exos displayed similar effects in an inflammatory model of OA, where BM-MSCs were already shown to exert a therapeutic function^2^. We injected 250 ng Exos or 500 ng MPs (equivalent of 48 h production by plated 2.5 × 10^5^ BM-MSCs) at day 7 after OA induction. At day 42 we evaluated cartilage degradation in the medial plateau, which is the most affected tibia part in the CIOA model, using CLSM analysis. We demonstrated a significant improvement of all parameters of articular cartilage, including volume, cartilage degradation (surface/volume ratio) and thickness in treated mice (Fig. 4A,B). No difference was observed between treated groups or healthy mice. These results were confirmed by histological analysis of tibias sections and OA scoring, which indicated protection of cartilage degradation for treated mice and nice reduction of osteophyte formation although significant only for Exos (Fig. 4C,D).

**Figure 5:**
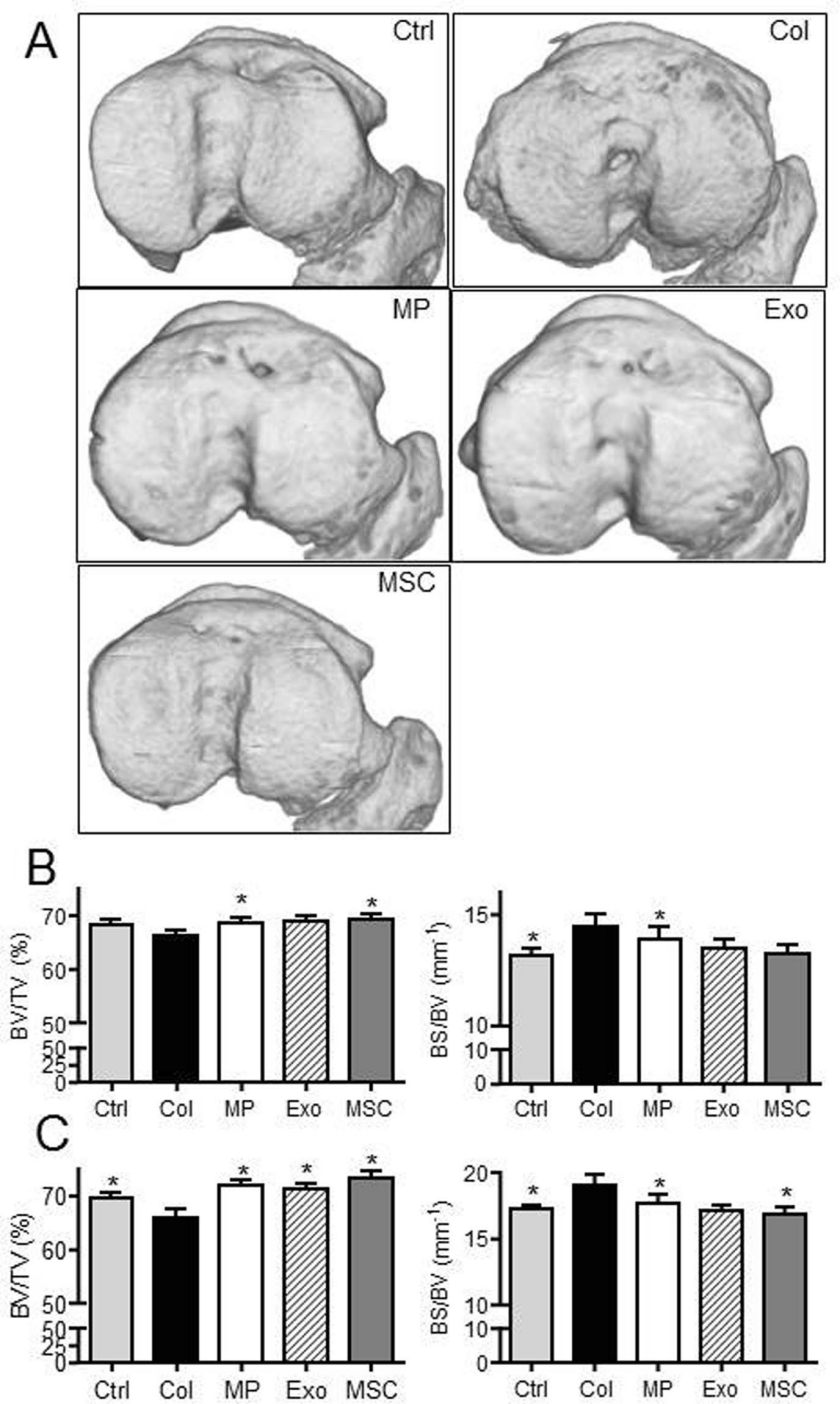
BM-MSC-derived MPs and Exos protected mice from osteoarthritic damages in the collagenase-induced OA model. (**A**) Representative 3D reconstructed images of sub-chondral bone surface in tibias after µCT analysis. Images from control mice (Ctrl), collagenase-treated mice (Col) and Col mice that received intra-articular injection of 500 ng MPs or 250 ng Exos or 2.5 × 10^5^ BM-MSCs. (**B**) Histomorphometric analysis of 3D images of epiphyseal bone: Bone volume/tissue volume (BV/TV) and bone surface/bone volume (BS/BV) parameters (n = 15). (**C**) Histomorphometric analysis of sub-chondral bone. *: p < 0.05 as compared to Col group.

**Figure 6:**
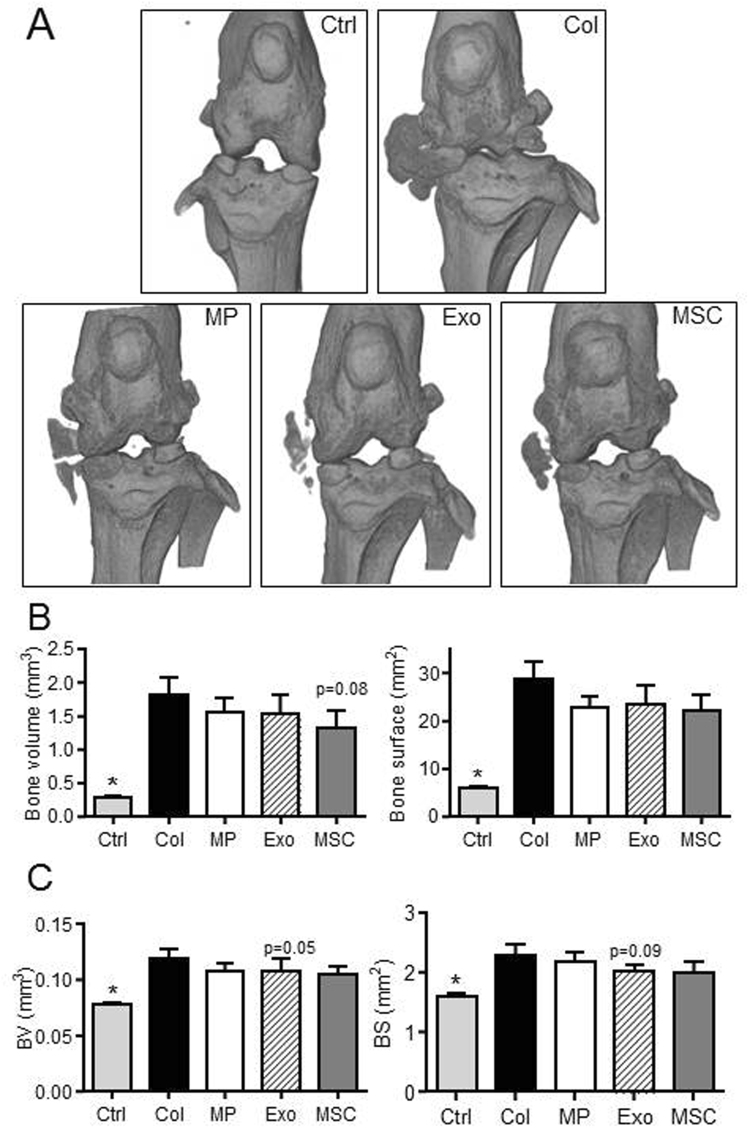
BM-MSC-derived MPs and Exos protected mice from osteoarthritic damages in the collagenase-induced OA model. Images from control mice (Ctrl), collagenase-treated mice (Col) and Col mice that received intra-articular injection of 500 ng MPs or 250 ng Exos or 2.5 × 10^5^ BM-MSCs. (**A**) Representative 3D reconstructed images of bone knee joints. (**B**) Bone volume and bone area as measured in the menisci and external ligaments. (**C**) Histomorphometric analysis of osteophytes at the edges of joint bone. *: p < 0.05 as compared to Col group.

We also assessed effect of treatments on histomorphometric parameters of bone by µCT. We evaluated bone parameters of epiphyseal bone and sub-chondral bone in the median part of treated and OA joints. At the epiphyseal level, we measured significantly higher bone volume (BV/TV parameter) in MP- and BM-MSC-treated mice as well as less bone degradation (BS/BV parameter) in MP-treated mice compared to OA control mice (Fig. 5A,B). At the sub-chondral bone levels, results were even more significant with higher bone volume and lower bone degradation for all treated mice as compared to OA controls (Fig. 5C). We also noticed calcification of the median ligaments and menisci in OA control mice that were not observed in healthy mice or to a lesser extent in treated mice (Fig. 6A). Indeed although not significant, bone volumes and bone areas of ligaments and menisci as well as osteophytes were lower in all treated mice, including MP-, Exos- and BM-MSC-treated joints (Fig. 6B,C). Altogether, accurate analyses of histomorphometric parameters of the entire articular cartilages and epiphyses of mice demonstrated a chondroprotective role of relatively low doses of both MPs and Exos isolated from BM-MSCs.

## Discussion

In the present study, we demonstrated that MPs and Exos isolated from adult bone marrow-derived murine BM-MSCs exert a similar chondroprotective effect in the collagenase-induced OA model. This is the first demonstration that extracellular vesicles generated from different cell compartments and pathways (exocytosis of exosomes from multivesicular bodies from the endosomal compartment or release of microparticles by cell membrane budding) exhibit a similar *in vivo* function in osteoarthritis.

We recently described the interest of using murine bone marrow BM-MSC-derived Exos or MPs to reduce clinical symptoms in the collagen-induced arthritis inflammatory model (Cosenza *et al*., manuscript in revision). In this study, we showed that both Exos and MPs were able to inhibit *in vitro* activation of CD4^+^ and CD8^+^ T lymphocytes and B lymphocytes. *In vivo*, clinical signs of arthritis were slow down following injection of Exos but not MPs through inhibition of plasmablast differentiation and IL-10 expressing Breg cell induction. Although this was the first study reporting the therapeutic efficacy of BM-MSC-derived Exos in inflammatory arthritis, previous studies demonstrated the interest of using Exos from genetically engineered dendritic cells or neutrophils-derived Exos in the collagen-induced arthritis model^19–22^.

Beneficial effect of BM-MSC-derived exosomes has been recently reported in the CIOA model using human synovium MSCs or iPS-derived MSCs^12^. The authors reported improvement of OARSI score and increased migration and proliferation potential of chondrocytes incubated with Exos. In the second publication, Exos from human synovial MSCs protected cartilage from degradation in a rat model of OA, although far less efficiently than Exos isolated from miR-140-5p-over-expressing MSCs^11^. A more recent publication described that exosomes from embryonic stem cells-derived mesenchymal stem cells impeded cartilage destruction in the Destabilization of Median Meniscus (DMM) model^13^. However, none of these studies compared the respective role of Exos and microparticles nor that of mixed populations of EVs. In concordance with those pre-clinical studies, we here demonstrated that adult BM-MSC-derived Exos could efficiently protect cartilage and bone from degradation. Using respectively, CLSM and µCT for histomorphometric analyses of 3D reconstructions of those tissues, accurate quantitative measures of the whole tissues were possible where standard histological sections of joints are only representative^23^. These analyses demonstrated that Exos and MPs were equally efficacious to protect mice from developing OA suggesting that both EVs share common mechanisms for cartilage and bone protection. Further investigation of the mechanism of action shared by these EVs is necessary. Very preliminary data from our laboratory indicated that MPs and Exos convey common miRNAs that warrant validation. We also quantified similar amounts of immunosuppressive molecules (PGE2, IL1-RA, TGFβ1) in both types of vesicles that could be involved in their anti-inflammatory effect (Cosenza *et al*., in revision).

Indeed, we demonstrated *in vitro* that both Exos and MPs from BM-MSCs exerted similar functions as the parental cells. They were able to reinduce the expression of markers of mature articular chondrocytes (type II collagen and aggrecan) while decreasing catabolic (MMP-13, ADAMTS5) and inflammatory (iNOS) markers, in a dose-dependent manner for several markers. Interestingly, the highest dose of MPs or Exos reversed the OA phenotype of chondrocytes to a similar extent as BM-MSCs that were cocultured onto a transwell membrane. BM-MSC-CM was efficient for increasing the expression of anabolic markers with no effect on catabolic markers. These data suggest that anti-catabolic mediators are primarily conveyed by EVs but likely present at low concentrations as soluble molecules in the culture supernatants. Another interesting finding is the role of pre-activating BM-MSCs by TGFβ3 to enhance the efficacy of Exos and MPs. Although the observed differences were rather low as compared to non-activated BM-MSCs, the modulation of expression levels of marker genes by MPs or Exos reached statistical significance after BM-MSC pre-activation. Interest of TGFβ3 pre-activation of BM-MSCs on OA chondrocyte phenotype is an unpublished observation from our laboratory (Ruiz *et al*., manuscript submitted). The role of TGFβ3 induction on the chondrogenic differentiation of BM-MSCs is largely described^24^. Moreover, its role in mediating the anti-fibrotic function of BM-MSCs has also been reported^25^. We may therefore speculate that TGFβ3 pre-activation of BM-MSCs stimulates their pro-chondrogenic and anti-fibroblastic function through the release of factors that might be conveyed by MPs and Exos. Our results on the beneficial effect of Exos and MPs on OA chondrocytes are not in line with those recently described using human OA chondrocytes and BM-MSC-derived Exos^11^. The authors described *in vitro* down-regulation of type II collagen, aggrecan and Sox9 but increased proliferation and migration of chondrocytes. The explanation for this discrepancy is not known but may be related to the use of human synovium-derived BM-MSCs that may display different functions than bone marrow-derived BM-MSCs. It is however more likely that Exos produced by synovial BM-MSCs recovered from patients conveyed inflammatory or catabolic mediators that were detrimental for chondrocytes. This is supported by a study showing that MPs derived from RA synovial fibroblasts contained high levels of ADAMTS5 promoting aggrecan destruction^26^. Another study reported hexosaminidase D activities in EVs from RA and OA patients that could contribute to cartilage degradation^27,28^. This pointed out the importance of using BM-MSCs isolated from healthy individuals for the prodution of MPs and Exos that could display beneficial and not detrimental effects on target cells or tissues.

We also showed that MPs and Exos protected chondrocytes from induced apoptosis, which is another feature of OA chondrocytes. Anti-apoptotic role of BM-MSC-derived Exos was already reported and recently reviewed for cardiovascular diseases^29^. Finally, we provided *in vitro* evidence that BM-MSC-derived MPs and Exos inhibited macrophage activation and some evidence of possible induction of a M2-like anti-inflammatory macrophage phenotype. Monocytes and macrophages, which are recruited in the synovial membrane via CCL2/CCR2 signalling, are thought to be main actors of inflammation and tissue damage by producing pro-inflammatory and catabolic mediators in OA^30,31^. We previously reported that BM-MSCs induced the polarisation of macrophages towards an anti-inflammatory phenotype thereby reducing the inflammatory activation of synovium^9,32^. Macrophage polarization induced by BM-MSCs was shown to be mediated by PGE2 and we have detected PGE2 in BM-MSC-derived MPs and Exos (Cosenza *et al*., in revision). We may therefore hypothesize that PGE2 conveyed by MPs and Exos isolated from BM-MSCs is at least partly responsible for inhibition of macrophage activation and possible M2 macrophage polarization *in vitro* and *in vivo* after IA injection. This is further supported by a recent study showing that IL1β-pretreated BM-MSCs could induce macrophage polarization toward a M2 phenotype more efficiently than naïve BM-MSCs and that miR-146a-containing Exos contributed to this effect^33^.

In conclusion, we provided evidence that MPs and Exos from murine bone marrow BM-MSCs exerted similar functional effect *in vitro* by re-establishing chondrocyte homeostatic state, protecting chondrocytes from apoptosis and stimulating macrophage polarization towards anti-inflammatory phenotype. All of these functions displayed by BM-MSC-derived MPs and Exos might explain their beneficial effect in the CIOA model where treated mice were partly protected from cartilage and bone degradation.

## Electronic supplementary material

Supplementary Figure 1
