# Supplementary material for: Mesenchymal stem cells derived exosomes and microparticles protect cartilage and bone from degradation in osteoarthritis
Source: Sci Rep. 2017 Nov 24;7:16214. doi: 10.1038/s41598-017-15376-8 (PMC5701135; doi:10.1038/s41598-017-15376-8)
Supplement: Supplementary file 1 — Supplementary Figure 1 [file 41598_2017_15376_MOESM1_ESM.pdf]

Supl Fig 1

Mesenchymal stem cells derived exosomes and microparticles protect cartilage and bone from degradation in osteoarthritis

Stella Cosenza[a], Maxime Ruiz[a], Karine Toupet[a], Christian Jorgensen[a,b,*], Danièle Noël[a,b,*]

[a]IRMB, INSERM, Montpellier University, Montpellier, France; [b]Clinical immunology and osteoarticular diseases Therapeutic Unit, Hôpital Lapeyronie, Montpellier, France
*: equally contributing authors

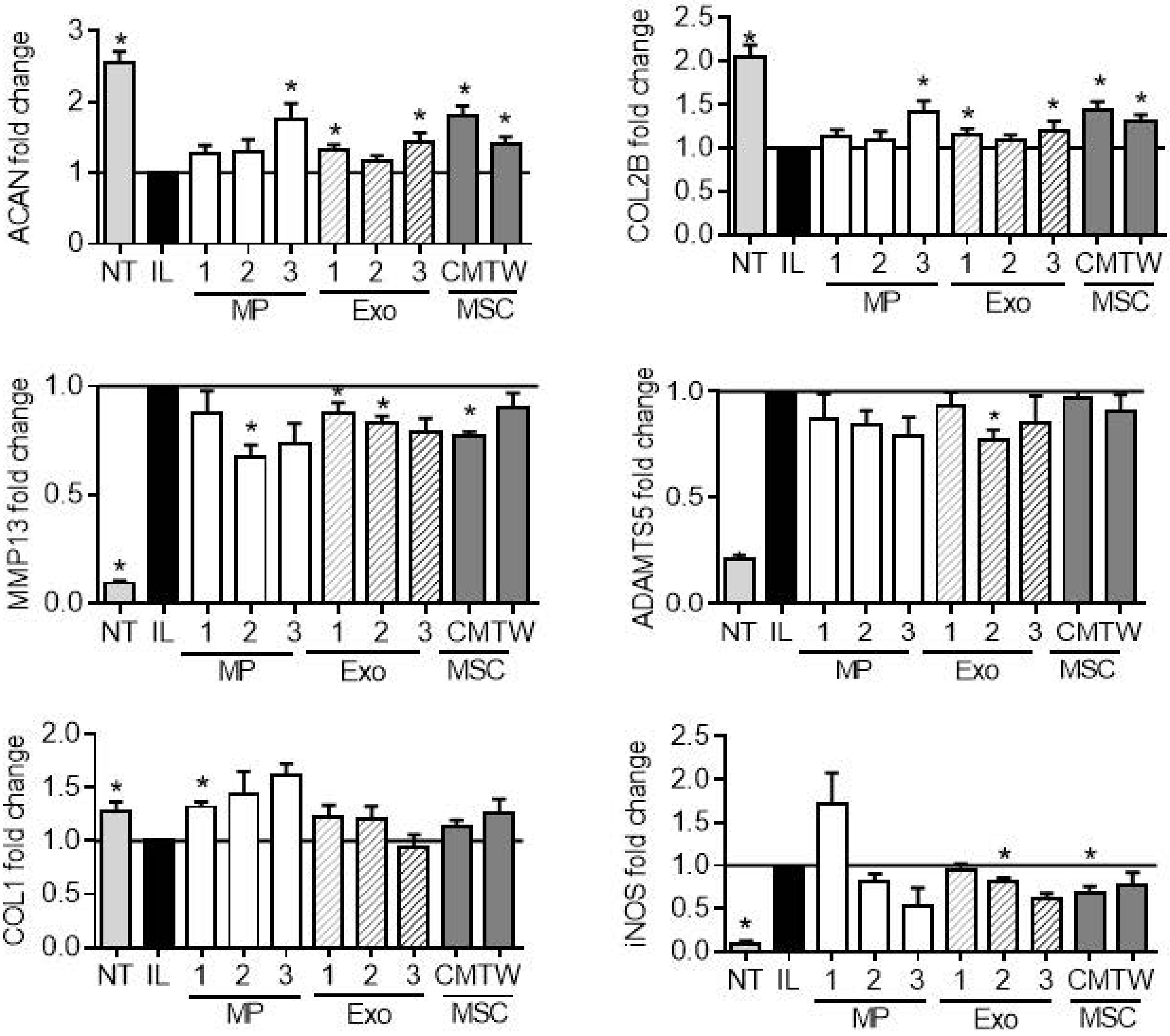

Supl Figure 1. BM-MSC-derived MPs and Exos exerted chondroprotective effect on OA-like chondrocytes. Primary murine chondrocytes were pretreated with 1 ng/mL IL-1β (IL) or not (NT) for 24h before addition of different amounts of MPs or Exos (1: 12.5 ng; 2: 125 ng; 3: 1.25 µg), 1 mL BM-MSC-CM (before centrifugation) or BM-MSCs (10^5 cells) on top of a transwell membrane (TW). Expression of chondrocyte markers was quantified by RT-qPCR after 24h (n=13). *: $p<0.05$ as compared to IL1-β-treated OA-like chondrocytes.
